# An Exploratory Study of Vitamin D and Matrix Metalloproteinase and Their Regulators in Polyendocrine Metabolic Ovarian Syndrome

**DOI:** 10.3390/ph19081144

**Published:** 2026-07-24

**Authors:** Mashael Zainalabedin, Nora Smahi, Thozhukat Sathyapalan, Alexandra E. Butler, Stephen L. Atkin

**Affiliations:** 1 School of Medicine, Royal College of Surgeons in Ireland-Medical University of Bahrain, Busaiteen 15503, Bahrain; mzainalabedin@rcsi-mub.com (M.Z.); 25204417@rcsi-mub.com (N.S.); satkin@rcsi.com (S.L.A.); 2 Hull York Medical School, University of Hull, Hull HU6 7RX, UK; thozhukat.sathyapalan@hyms.ac.uk

**Keywords:** Polyendocrine Metabolic Ovarian Syndrome (PMOS), vitamin D, MMP, TIMP, 25 hydroxyvitamin D_3_, 1,25 dihydroxyvitamin D_3_

## Abstract

**Objective:** Vitamin D has been reported to act as a tissue-protective regulator by suppressing Matrix Metalloproteinases (MMPs) and upregulating Tissue Inhibitors of Metalloproteinases (TIMPs), though changes may be body mass index (BMI)-dependent; however, the relationship between vitamin D metabolism and matrix remodeling pathways in Polyendocrine Metabolic Ovarian Syndrome (PMOS) remains poorly understood. **Methods:** In women with PMOS (*n* = 28) and controls (*n* = 28), 12 MMPs and 3 TIMPs were determined by Slow Off-rate Modified Aptamer (SOMA)-scan plasma protein measurement and correlated to 25-hydroxyvitamin D_3_ (25(OH)D_3_) and its metabolites (active 1,25-dihydroxyvitamin D_3_ (1,25(OH)_2_D_3_) and 24,25-dihydroxyvitamin D_3_ (24,25(OH)_2_D_3_)) measured by gold standard isotope-dilution liquid chromatography tandem mass spectrometry. **Results:** Insulin resistance and systemic inflammation (normal C-reactive protein) were comparable between PMOS and control women, though PMOS had higher free androgen index and anti-Mullerian hormone levels. Vitamin D and its metabolites did not differ between groups. Only MMP16 was lower in PMOS than controls (549.6 ± 58.3 vs. 678.0 ± 304.1 Relative Fluorescent Units, *p* = 0.037) but did not pass the false discovery rate. In women with PMOS, 25(OH)D_3_ and 24,25(OH)_2_D_3_ demonstrated significant inverse correlations with TIMP3 (r = −0.60, *p* = 0.005 and r = −0.58, *p* = 0.007, respectively). Multivariable regression confirmed independent inverse associations between TIMP3 and both 25(OH)D_3_ (β = −51.1, *p* = 0.031) and 24,25(OH)_2_D_3_ (β = −858.7, *p* = 0.028) after adjustment for age, body mass index and Homeostatic Model Assessment-Insulin Resistance, and the association remained stable following bootstrap internal validation. Additional moderate associations were observed between vitamin D metabolites and membrane-type MMPs (MMP14, MMP16, MMP17). **Conclusions:** Exploratory analyses suggested potential inverse associations between vitamin D metabolites and TIMP3, together with weaker associations involving MMP14, MMP16 and MMP17, suggesting the novel hypothesis that vitamin D may influence extracellular matrix remodeling and ovarian stromal biology through regulation of TIMP3-dependent pathways independently of obesity and insulin resistance, rather than through MMPs directly.

## 1. Introduction

Polyendocrine Metabolic Ovarian Syndrome (PMOS; formerly polycystic ovary syndrome, PCOS) is the name recently agreed upon by international consensus, adopted to reflect the endocrine, metabolic and ovarian dysfunction of the condition and to avoid the implication of ovarian cysts conveyed by the former term [1]. PMOS is a common endocrine disorder of women of reproductive age and is the most prevalent endocrine abnormality in premenopausal women [2]. It is diagnosed using recognized international criteria after exclusion of other conditions [3], and an estimated 70% of affected women remain undiagnosed [4].

Beyond its reproductive features, PMOS carries substantial metabolic risk; affected women show higher rates of hypertension, dyslipidemia, type 2 diabetes and other cardiovascular risk factors [5]. Hyperandrogenemia, insulin resistance (IR), oxidative stress, chronic inflammation and vitamin D insufficiency are each implicated in the syndrome and are interrelated, so that their individual contributions are difficult to separate [6,7]. This complicates interpretation of circulating biomarkers, as markers such as heat shock proteins [8], complement and coagulation proteins [9] are altered in PMOS but are driven largely by coexisting obesity and IR [10,11,12,13]. Disturbances intrinsic to the syndrome are therefore difficult to distinguish from those secondary to obesity and IR.

Vitamin D synthesis initiates in the skin, where ultraviolet B radiation (UV-B) converts 7-dehydrocholesterol to vitamin D_3_ (cholecalciferol), with subsequent steps in the liver to 25-hydroxyvitamin D_3_ (25(OH)D_3_), and in the kidney to the active hormone 1,25(OH)_2_D_3_. 24,25-dihydroxyvitamin D_3_ (24,25(OH)_2_D_3_) is a measured metabolite in this pathway [14]. Levels of 1,25(OH)_2_D_3_ are also contributed to by extra-renal macrophages through the type 2 interferon response, whose regulation differs from that of the kidney [15]. Vitamin D acts via binding to the vitamin D receptor (VDR) and heterodimerization with the retinoid X receptor [16], and through binding to the membrane VDR or the 1,25D-membrane-associated, rapid-response steroid-binding protein receptor [17]. The bound 1,25(OH)_2_D_3_–VDR complex regulates transcription of the insulin gene [18].

**Figure 1:**
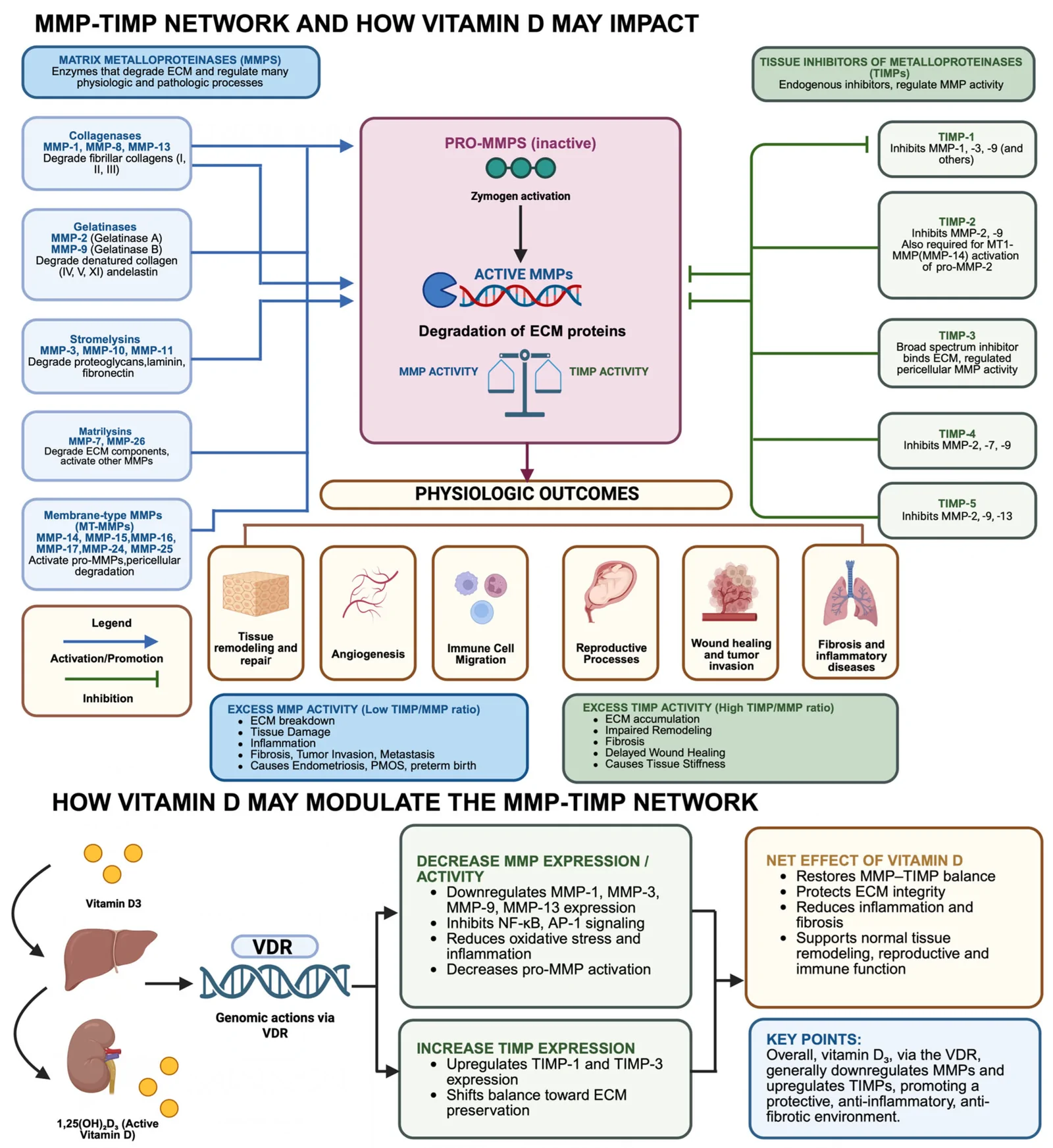
An overview of the Matrix Metalloproteinase -Tissue Inhibitors of Metalloproteinases network, including the function of subsets of MMPs on the ECM, primarily collagenases, gelatinases, stromelysins, matrilysins, and membrane-type MMPs, as well as the inhibitory roles of TIMP-1, -2, -3, -4 and -5 on their respective MMPs. Balance between MMP and TIMP activity regulates various physiologic outcomes, including angiogenesis, reproductive processes, tissue remodeling and repair, immune cell migration, wound healing and tumor invasion, as well as fibrosis and inflammatory diseases. Therefore, imbalance between MMP and TIMP activity can result in various disease states. In High MMP/Low TIMP conditions, inflammation, tissue injury, PMOS, endometriosis, cancer metastasis and preterm birth can occur, whilst fibrosis, impaired wound healing and tissue stiffness can be symptomatic of Low MMP/High TIMP levels. Vitamin D_3_ is proposed as a regulator for High MMP/Low TIMP states. Vitamin D_3_, transported to the liver from the skin and then to the kidneys where it is activated, can modulate the MMP-TIMP network, as it upregulates TIMP-1 and TIMP-3 expression, which in turn downregulates MMP-1, -3, -9, -13 levels, reducing inflammation and ultimately shifting the balance towards ECM preservation. Thus, homeostasis of tissue remodeling processes, reproductive and immune function can be restored. Matrix Metalloproteinase (MMP); Tissue Inhibitors of Metalloproteinases (TIMP); ECM, extracellular matrix; VDR, Vitamin D receptor; Nuclear Factor kappa B (NF-κB); Activator Protein-1 (AP-1). Created in BioRender. Butler, A. (2026) https://BioRender.com/i31dvb3 (accessed on 1 July 2026).

Low vitamin D status accompanies obesity, higher IR and raised testosterone, characteristics frequently found in PMOS [19,20], and vitamin D replacement has been reported to affect IR and steroidogenesis in affected obese women [21]. Severe deficiency is frequent, reported in 67–85% of affected women [21,22], and because vitamin D is sequestered in adipose tissue, its circulating concentration falls with increasing obesity, so the deficiency associated with IR is itself affected by adiposity [23]. Vitamin D has further roles in inflammation, oxidative-stress regulation and mitochondrial function, with antioxidant activity [24], and these actions may be independent of obesity [25]. Whether 25(OH)D_3_ is anti-inflammatory is uncertain, with some studies reporting an effect and others none [26], although supplementation has been shown to reduce C-reactive protein (CRP) and stabilize interleukin-10 [27]; the anti-inflammatory actions of 1,25(OH)_2_D_3_, mediated through macrophage and T-cell responses, are more clearly established [28,29,30] (Figure 1). In addition, vitamin D interacts with matrix remodeling pathways, including the MMP/TIMP axis, and it is reported that vitamin D suppresses MMP activity in chronic disorders, as well as in human uterine fibroid cells [31,32] (Figure 1).

Matrix metalloproteinases (MMPs) are the principal enzymes that degrade extracellular matrix (ECM) proteins during tissue remodeling [33], and their activity is regulated by the tissue inhibitors of metalloproteinases (TIMPs). Because remodeling reflects the balance between proteases and their inhibitors, the ratio of an MMP to its regulating TIMP may provide information on ECM turnover, and such ratios have been proposed as biomarkers of disease states [34]. MMP activity has been implicated in the pathogenesis of PMOS [35] and, within the ovary [36], both MMPs and TIMPs contribute to normal physiology, including follicular rupture and oocyte release, as well as to pathophysiology [37].

Reported MMP and TIMP findings in PMOS are inconsistent. An imbalance with reduced TIMP2 and increased MMP2/TIMP2 and MMP9/TIMP1 ratios has been described [35], yet circulating MMP2 and MMP9 have been reported as elevated [38,39,40], unchanged [35], or reduced [41]. Much of this variation appears to be related to body mass index (BMI), with which these proteases generally correlate, although increased MMP9 has also been reported independently of BMI [42,43]. As obesity and IR are closely associated with PMOS, it is unclear whether vitamin D metabolites relate to the MMP/TIMP system through the syndrome itself or through the accompanying obesity and insulin resistance.

Studying women with PMOS who are neither obese nor insulin-resistant allows these two confounders to be removed rather than adjusted for statistically. We therefore correlated vitamin D_3_ (25(OH)D_3_) and its metabolites with circulating MMP and TIMP concentrations in non-obese, non-IR women with PMOS and in age- and BMI-matched controls without PMOS. The null hypothesis was that 25(OH)D_3_ would not correlate with MMPs or TIMPs and that, after accounting for these parameters, MMP and TIMP concentrations would not differ between the non-obese PMOS and control groups.

## 2. Results

Demographic, hormonal and metabolic data for the 28 PMOS subjects and 28 controls are shown in Table 1. The two cohorts were weight-, IR- and age-matched with no evidence of increased systemic inflammation (CRP was not elevated and did not differ between the groups), but subjects with PMOS did have hyperandrogenemia and elevated anti-Müllerian hormone (AMH). 25(OH)D_3_, the active form 1,25(OH)_2_D_3_ and the inactive form 24,25(OH)_2_D_3_ did not differ between groups (Table 1).

Among the MMP and TIMP markers examined, only MMP16 was significantly lower in women with PMOS than controls (549.6 ± 58.3 vs. 678.0 ± 304.1 Relative Fluorescent Units (RFU), *p* = 0.037); this association did not remain significant following correction for multiple testing. No significant differences were observed for the remaining MMPs or TIMPs (Table 2).

Correlation analysis showed that there was an inverse association between 25(OH)D_3_ and TIMP3 (r = −0.60, *p* = 0.005), and a positive association between 25(OH)D_3_ and MMP16/TIMP3 r = 0.54, *p* = 0.017) and between 25(OH)D_3_ and MMP17/TIMP3 (r = 0.6, *p* = 0.006) (Table 3). There was a negative association between 24,25(OH)_2_D_3_ and TIMP3 (r = −0.58, *p* = 0.007), but positive associations between 24,25(OH)_2_D_3_ and MMP14/TIMP3 (r = 0.45, *p* = 0.049), 24,25(OH)_2_D_3_ and MMP16/TIMP3 (r = 0.53, *p* = 0.015) and 24,25(OH)_2_D_3_ and MMP17TIMP3 (r = 0.59, *p* = 0.006) (Table 3).

A trend to significance was seen for 1,25(OH)_2_D_3_ and MMP14 (r = −0.464, *p* = 0.061); 25(OH)D_3_ and MMP17 (r = 0.44, *p* = 0.052); 25(OH)D_3_ and MMP16 (r = 0.397, *p* = 0.083); 24,25(OH)_2_D_3_ and MMP17 (r = 0.419, *p* = 0.066) (Supplementary Table S1).

In multivariable regression analyses of the combined cohort (*n* = 48), adjusting for age, BMI, and HOMA-IR, both 25(OH)D_3_ and 24,25(OH)_2_D_3_ were inversely associated with circulating TIMP3. Each 1-unit increase in 25(OH)D_3_ was associated with a 51.1-unit reduction in TIMP3 (β = −51.1; 95% CI −97.2 to −5.0; *p* = 0.031). This association remained stable following bootstrap internal validation (bootstrap 95% CI −93.6 to −12.6; bootstrap *p* = 0.010). Each 1-unit increase in 24,25(OH)_2_D_3_ was associated with an 858.7-unit reduction in TIMP3 (β = −858.7; 95% CI −1617.9 to −99.5; *p* = 0.028), with a bootstrap 95% CI of −1556.0 to −227.7 (bootstrap *p* = 0.005) (Table 4).

Age, BMI, and HOMA-IR were not independently associated with TIMP3 in either model. However, the overall explanatory power of the models was modest: the 25(OH)D_3_ model had an R^2^ of 0.116 and an adjusted R^2^ of 0.034, while the 24,25(OH)_2_D_3_ model had an R^2^ of 0.120 and an adjusted R^2^ of 0.038. These findings should therefore be interpreted as exploratory associations rather than evidence of a robust predictive model.

## 3. Discussion

The PMOS patients recruited for this study were matched to controls for age, BMI, IR and CRP, as well as having equivalent levels of 25(OH)D_3_, 1,25(OH)_2_D_3_ and 24,25(OH)_2_D_3_. Our hypothesis was that such matched groups would have no difference in systemic MMP and TIMP levels, nor differences in MMP/TIMP ratios, between cohorts. Only MMP16 was lower in PMOS compared to controls; however, this did not survive FDR testing. The absence of robust differences in circulating MMPs and TIMPs, following multiple-testing correction in PMOS compared to controls, suggests that there was no systemic dysregulation of the MMP/TIMP axis in this cohort.

TIMP3 is unique among the tissue inhibitors of metalloproteinases because it is tightly bound to the extracellular matrix and regulates not only matrix metalloproteinases but also members of the A Disintegrin and Metalloproteinase (ADAM) family, particularly ADAM17/tumor necrosis factor-α converting enzyme (TACE) [44,45] Through these actions, TIMP3 influences ECM turnover, inflammatory signaling, epidermal growth factor receptor activation, angiogenesis, fibrosis and tissue remodeling [44,45,46]. Experimental studies suggest that TIMP3 plays an important role in ovarian physiology through regulation of follicular development, ovulation, and stromal remodeling, processes that are known to be altered in women with PMOS [37,47]. Furthermore, TIMP3 has been implicated in adipose tissue inflammation, insulin sensitivity, and hepatic fibrosis, linking extracellular matrix regulation to the broader metabolic disturbances associated with PMOS [44]. Circulating TIMP3 levels may not reflect tissue TIMP3 processes in the ovary; however, the observed inverse association between vitamin D metabolites and TIMP3 in this study raises the possibility that vitamin D may influence ovarian ECM homeostasis through mechanisms extending beyond classical MMP inhibition. While these findings require validation in larger cohorts, they suggest a potentially novel interaction between vitamin D metabolism and TIMP3-dependent tissue remodeling pathways in PMOS. Surprisingly, Vitamin D metabolites were found to independently and inversely associate with TIMP3 in the PMOS subjects, after adjustment for BMI and HOMA-IR. The strongest associations observed were inverse correlations between the vitamin D metabolites 25(OH)D_3_ and 24,25(OH)_2_D_3_ and TIMP3; moreover, vitamin D metabolites were positively correlated with MMP16/TIMP3. These findings were only present in the PMOS cohort; however, as circulating vitamin D concentrations themselves did not differ between PMOS and controls, this suggests that the observed relationships were not fully explained by adiposity or insulin resistance. Instead, it could be postulated that PMOS may not be characterized by major alterations in circulating vitamin D or extracellular matrix remodeling proteins individually, but rather by altered interactions between vitamin D metabolism and matrix remodeling pathways. The potential correlation of 24,25(OH)_2_D_3_ with TIMP3 is interesting as 24,25(OH)_2_D_3_ is regarded as an inactive metabolite in the vitamin D_3_ metabolic pathway; however, 24,25(OH)_2_D_3_ may not be an inactive metabolite [48], and it may have a physiological role in growth [49] and in dyslipidemia [50].

Previously, studies have linked vitamin D status with metabolic disturbances, including obesity and insulin resistance, in women with PMOS, but not necessarily with the condition itself [19]. It is reported that vitamin D interacts with matrix remodeling pathways, including the MMP/TIMP axis, and vitamin D suppresses MMP activity in chronic disorders, as well as in human uterine fibroid cells [31,32]. Balanced MMP/TIMP activity is fundamental for normo-ovarian physiology to enable follicular rupture and oocyte release [37]. Therefore, disturbances in the balance between MMP/TIMP activity may lead to the dysregulation of ovarian ECM remodeling and increased MMP activity, which has been reported for MMP9, which has been previously associated with the dysregulation of ECM remodeling in women with PMOS [51]. Additionally, higher MMP9/TIMP3 ratios have recently been identified in PMOS patients [43].

A preliminary hypothesis for the findings of this study would be that there is a novel Vitamin D-TIMP3 correlation in the context of PMOS. The inverse associations between vitamin D metabolites and circulating TIMP3 concentrations in the PMOS cohort reported, independent of BMI and HOMA-IR, may suggest that vitamin D interacts with ECM regulatory pathways in PMOS, a feature that has not been previously reported. No significant differences in circulating MMP and TIMP levels were observed between the control and PMOS cohorts, which suggests that systemic ECM remodeling is likely preserved in PMOS. Thus, it is possible that abnormalities in matrix remodeling are localized at the ovarian or endometrial tissue levels and are not reflected in peripheral blood measurements. TIMP3 is expressed in the liver, lungs, inflammatory cells, and many other tissues beyond reproductive tissue [44]; hence, it cannot be assumed that circulating TIMP3 levels originate from the ovary alone; however, the absence of differences in circulating TIMP3 does not exclude localized dysregulation within the ovarian microenvironment.

A key strength of this study included the investigation of multiple vitamin D metabolites, identifying that circulating vitamin D and its metabolites did not differ between PMOS and matched controls, suggesting that global vitamin D metabolism was not disrupted in this cohort [52]. The cohort was closely matched to controls, allowing comparison of a panel of proteins without the bias of age, BMI, inflammation and IR. Blood samples were taken on day 21 of the menstrual cycle to limit any additional fluctuations due to changes in the menstrual cycle. Limitations include the modest number of subjects in each group, and there was a lack of ethnic diversity as the study was performed on an exclusively Caucasian population. The effect of hormonal stimulation prior to the 9-month washout period having a residual effect cannot be excluded. The exploratory study population was all undergoing IVF therapy, and a larger study in a broader population is needed to validate the results. Circulating measurements may not reflect ovarian or endometrial tissue concentrations; hence, there is a need for follicular fluid or endometrial tissue analyses. Further molecular-level analysis is required within a larger study to validate the potential role of vitamin D and its metabolites on tissue restructuring through TIMP regulation.

## 4. Materials and Methods

### Study Design

Plasma MMP and TIMP levels were determined in 28 women with PMOS and 28 control women matched for age and BMI (<29 kg/m^2^), recruited from the Hull in vitro fertilization (IVF) clinic [14]. A total of 36 women with PMOS who met those criteria were approached, of whom 28 agreed to participate. Sequential control women were then recruited within the age range of the PMOS: 31 were approached, of whom 28 agreed to participate. The study was approved by the Yorkshire and The Humber NRES ethical committee, UK (ethics number 02/03/043), and was performed in accordance with the approved ethical protocol.

PMOS was diagnosed using Rotterdam consensus criteria, which included biochemical hyperandrogenemia, defined as free androgen index (FAI) >4, clinical hyperandrogenism, defined as a Ferriman-Gallwey score >8, amenorrhea or oligomenorrhea, and polycystic ovaries identified through transvaginal ultrasound [53]. All participants were otherwise healthy and had not taken prescription or over-the-counter medication for ≥9 months before enrollment. Endocrine causes that may mimic PMOS were excluded by appropriate testing, namely non-classical 21-hydroxylase deficiency, Cushing’s disease, androgen-secreting tumors, and hyperprolactinemia [3]. Table 1 summarizes the baseline demographic and biochemical profile of both groups.

Subjects had a fasting blood sample that was taken in the luteal phase of the cycle (day 21) prior to initiating IVF treatment. Height, weight and waist circumference were recorded according to World Health Organization (WHO) recommendations [54], and BMI was calculated as kg/m^2^. Plasma was separated from fasting blood samples by centrifugation at 3500 g for 15 min, aliquoted and stored at −80 °C until analysis. Sex hormone-binding globulin (SHBG) and insulin were measured using a DPC Immulite 200 analyzer (Euro/DPC, Llanberis, UK). Plasma glucose, used for calculation of homeostasis model assessment-insulin resistance (HOMA-IR), and C-reactive protein (CRP) were measured using a Synchron LX20 analyzer (Beckman-Coulter, High Wycombe, UK). The FAI was derived as the ratio of total testosterone to SHBG multiplied by 100. Testosterone was quantified in serum by isotope-dilution liquid chromatography tandem mass spectrometry (LC-MS/MS) [14], with vitamin D and its metabolites assayed by the same isotope-dilution LC-MS/MS approach [55].

Plasma MMPs and TIMPs were quantified in EDTA plasma on the Slow Off-rate Modified Aptamer (SOMAscan) platform [56], following previously validated protocols [57,58]. An overview of the protocol involves the incubation of plasma samples with synthetic fluorophore-labeled SOMAmer reagents, linked to biotin through a photocleavable linker, to bind target analytes. The analyte-SOMAmer complexes were captured on a streptavidin-substituted matrix and released into solution by ultraviolet cleavage of the photocleavable linker. The complexes were then immobilized through analyte-associated biotinylation on a streptavidin matrix. After elution, the released SOMAmers were used as quantitative surrogates for the corresponding analytes, with quantification performed by hybridization to complementary oligonucleotides. Calibration relied on assay reference standards [59]. Raw intensities underwent standardization and normalization for hybridization, median signal and calibration signal [59].

All measurements were performed on SOMAscan Version 3.1, which covered 12 MMPs (MMP1, MMP2, MMP3, MMP7, MMP8, MMP9, MMP10, MMP12, MMP13, MMP14, MMP16 and MMP17) and 3 TIMPs (TIMP1,2,3), as listed in Table 2.

## 5. Statistics

This was an exploratory study as there were no previous studies on the MMP and TIMP panel and their relationship to vitamin D to allow for a formal power calculation. Data were assessed for normality using the Shapiro–Wilk test and visual inspection of histograms and Q–Q plots. Continuous variables are presented as mean ± standard deviation (SD), while categorical variables are presented as frequencies and percentages.

Comparisons between women with PMOS and healthy controls were performed using Welch’s independent samples *t*-test. Effect sizes were calculated using Cohen’s d with 95% confidence intervals (CI) and interpreted as small (0.2), medium (0.5) and large (0.8) according to conventional thresholds.

To provide a distribution-free assessment of group differences, permutation testing (10,000 permutations) was performed for all primary comparisons. Given the large number of biomarkers evaluated, adjustment for multiple testing was undertaken using the false discovery rate (FDR) procedure, with adjusted q-values reported alongside nominal *p*-values.

Associations between vitamin D metabolites and MMPs or TIMPs were assessed using Pearson correlation coefficients. Correlation analyses were conducted separately in women with PMOS, controls, and the combined cohort. To account for multiple correlation testing, FDR correction was applied.

To determine whether observed associations were independent of adiposity, analyses of covariance (ANCOVA) were performed with BMI included as a covariate. Additional models incorporating insulin resistance, assessed by HOMA-IR, were explored where appropriate. Adjusted estimated marginal means and corresponding *p*-values are reported.

All statistical tests were two-tailed, and statistical significance was defined as *p* < 0.05. Statistical analyses were performed using SPSS version 26 (IBM Corp., Armonk, NY, USA).

## 6. Conclusions

Exploratory analyses suggest potential inverse associations between vitamin D metabolites and TIMP3 together with weaker associations involving MMP14, MMP16 and MMP17. These findings suggest the novel hypothesis that vitamin D may influence ECM remodeling and ovarian stromal biology through regulation of TIMP3-dependent pathways independently of obesity and IR, rather than through MMPs directly.

## Figures and Tables

**Table 1:** Demographics, baseline, hormonal and metabolic parameters of the PMOS subjects and controls (mean ± SD).

	Control (*n* = 28)	PMOS (*n* = 28)	*p*-Value
Age (years)	32.5 ± 4.1	31 ± 6.4	0.14
BMI (kg/m^2^)	24.8 ± 1.1	25.9 ± 1.8	0.56
Fasting glucose (nmol/L)	4.9 ± 0.4	4.7 ± 0.8	0.06
HbA1C (mmol/mol)	30.9 ± 6.5	31.8 ± 3.0	0.9
HOMA-IR	1.7 ± 1.0	1.6 ± 1.6	0.97
SHBG (nmol/L)	104 ± 80	72 ± 62	0.01
Thyroid stimulating hormone	1.9 ± 11.1	3.0 ± 3.5	0.26
17α-Hydroxyprogesterone (nmol/L)	6.5 ± 3.5	7.5 ± 4.5	0.42
Prolactin (mIU/L)	294 ± 109	352 ± 126	0.11
Free androgen index (FAI)	1.3 ± 0.5	4.1 ± 2.9 ± 4.0	0.0001
Ferriman-Galway score	3.4 ± 1.2	12.3 ± 2.5	0.0001
CRP (mg L^−1^)	2.3 ± 2.3	2.7 ± 2.5	0.43
AMH (ng/mL)	24.3 ± 13.1	57.2 ± 14.2	0.0001
25OHD_3_ (ng/mL)	22.7 (11.7–33.2)	21.8 (15.6–30.5)	0.89
1,25(OH)_2_D_3_ (ng/mL)	0.04 (0.03–0.05)	0.05 (0.035–0.06)	0.53
24,25(OH)_2_D_3_ (ng/mL)	1.02 ± 0.65	1.08 ± 0.68	0.73

BMI—Body Mass Index; HbA1c—glycated hemoglobin; HOMA-IR—Homeostasis model of assessment-Insulin Resistance; CRP—C-reactive protein; SHBG—sex hormone-binding globulin; AMH—Anti-Müllerian hormone. 25OHD_3_—25 hydroxyvitamin D_3_; 1,25(OH)_2_D_3_—1,25dihydroxyvitamin D_3_; 24,25(OH)_2_D_3_—24,25-dihydroxyvitamin D_3_.

**Table 2:** Comparison of circulating matrix metalloproteinases (MMP) and Tissue Inhibitors of Metalloproteinases (TIMP) Between Women with PMOS and Controls. Plasma concentrations of proteins are reported in Relative Fluorescent Units (RFU).

Marker	PCOS Mean ± SD	Control Mean ± SD	*p* (Welch)	Cohen’s d	d 95% CI Low	d 95% CI High
MMP-1	797 ± 385	979 ± 769	0.287	−0.28	−0.76	0.19
MMP-2	6525 ± 1498	6541± 1648	0.973	−0.01	−0.48	0.46
MMP-3	836 ± 1517	426 ± 118	0.243	0.42	−0.05	0.89
MMP-7	906± 290	1019 ± 301	0.197	−0.38	−0.85	0.09
MMP-8	2722 ± 1293	3493 ± 2914	0.222	−0.32	−0.79	0.15
MMP-9	26,265± 15,526	32,554 ± 19,392	0.219	−0.35	−0.82	0.12
MMP-10	756 ± 274	749± 330	0.940	0.02	−0.45	0.49
MMP-12	785 ± 247	887 ± 411	0.291	−0.29	−0.76	0.18
MMP-13	490± 134	681 ± 708	0.174	−0.35	−0.82	0.12
MMP-14	929± 377	1425 ± 1831	0.173	−0.35	−0.82	0.12
MMP-16	549 ± 58	678 ± 304	0.037	−0.54	−1.02	−0.07
MMP-17	711 ± 216	971± 829	0.123	−0.40	−0.87	0.07
TIMP-1	39.4 ± 7.3	57.5 ± 60.1	0.126	−0.39	−0.86	0.08
TIMP-2	320± 55.2	831 ± 226	0.317	−0.25	−0.72	0.22
TIMP-3	1932± 1740	1991 ± 1534.9	0.905	−0.04	−0.50	0.43

Values are mean ± SD. *p*-values derived from Welch’s *t*-test. Only MMP16 differed significantly between groups in unadjusted analyses; however, this association did not remain significant following correction for multiple testing.

**Table 3:** Significant Pearson correlation coefficients between Vitamin D Metabolites and matrix metalloproteinases (MMP) and Tissue Inhibitors of Metalloproteinases (TIMP) markers in women with PMOS. No correlations in controls. None survived FDR testing.

Vitamin D Variable	Target	Pearson r	Pearson *p*	Permutation *p*
25(OH)D_3_	TIMP3	−0.60	0.005	0.005
25(OH)D_3_	MMP16/TIMP3	0.54	0.014	0.017
25(OH)D_3_	MMP17/TIMP3	0.60	0.005	0.006
24,25(OH)_2_D_3_	TIMP3	−0.58	0.007	0.007
24,25(OH)_2_D_3_	MMP14/TIMP3	0.45	0.046	0.049
24,25(OH)_2_D_3_	MMP16/TIMP3	0.53	0.016	0.015
24,25(OH)_2_D_3_	MMP17/TIMP3	0.59	0.006	0.006

**Table 4:** Multivariable Linear Regression Models Examining Associations Between Vitamin D Metabolites and circulating TIMP3 concentrations. Model 1. TIMP3 as Dependent Variable; 25(OH)D_3_ as Primary Predictor. Model 2. TIMP3 as Dependent Variable; 24,25(OH)_2_D_3_ as Primary Predictor.

Predictor	β Coefficient	95% CI	*p*	Bootstrap β (Median)	Bootstrap 95% CI	Bootstrap *p*	Model R^2^	Adjusted R^2^
Model 1							0.116	0.034
25(OH)D_3_ (ng/mL)	−51.1	−97.2 to −5.0	0.031	−50.8	−93.6 to −12.6	0.01		
Age (years)	NS	—	>0.05	—	—	—		
BMI (kg/m^2^)	NS	—	>0.19	—	—	—		
HOMA-IR	NS	—	>0.19	—	—	—		
Model 2							0.12	0.038
24,25(OH)_2_D_3_ (ng/mL)	−858.7	−1617.9 to −99.5	0.028	−846.2	−1556.0 to −227.7	0.005		
Age (years)	NS	—	>0.05	—	—	—		
BMI (kg/m^2^)	NS	—	>0.19	—	—	—		
HOMA-IR	NS	—	>0.19	—	—	—		

Values represent regression coefficients (β) from multivariable linear regression models with TIMP3 as the dependent variable. Model 1 included 25(OH)D_3_, age, BMI and HOMA-IR. Model 2 included 24,25(OH)_2_D_3_, age, BMI and HOMA-IR. Bootstrap estimates were obtained using 10,000 non-parametric resamples. NS, not statistically significant. BMI, body mass index; HOMA-IR, homeostatic model assessment of insulin resistance.

## Data Availability

All the data for this study will be made available upon reasonable request to the corresponding author.

## Supplementary Materials

The following supporting information can be downloaded at: https://www.mdpi.com/article/10.3390/ph19081144/s1, Table S1: All Pearson correlation coefficients between Vitamin D Metabolites and matrix metalloproteinases (MMP) and Tissue Inhibitors of Metalloproteinases (TIMP) markers in women with PMOS. This includes the significant correlations presented in Table 3. No correlations in controls. None survived FDR testing.
