# Supplementary material for: An Exploratory Study of Vitamin D and Matrix Metalloproteinase and Their Regulators in Polyendocrine Metabolic Ovarian Syndrome
Source: Pharmaceuticals (Basel). 2026 Jul 24;19(8):1144. doi: 10.3390/ph19081144 (PMC13516373; doi:10.3390/ph19081144)
Supplement: Supplementary file 1 [file pharmaceuticals-19-01144-s001.zip › pharmaceuticals-4417909-supplementary.pdf]

Supplemental Table S1. All Pearson correlation coefficients between Vitamin D Metabolites and matrix metalloproteinases (MMP) and Tissue Inhibitors of Metalloproteinases (TIMP) markers in women with PMOS. This includes the significant correlations presented in Table 3. No correlations in controls. None survived FDR testing.

Group	Vitamin D variable	Target	Pearson r	Pearson p	Permutation p	FDR q
PMOS	$1,25(OH)_2D_3$	MMP9	-0.055	0.833	0.835	0.969
PMOS	$1,25(OH)_2D_3$	MMP3	0.446	0.073	0.061	0.510
PMOS	$1,25(OH)_2D_3$	MMP7	-0.144	0.581	0.597	0.927
PMOS	$1,25(OH)_2D_3$	MMP17	0.193	0.458	0.462	0.904
PMOS	$1,25(OH)_2D_3$	MMP2	0.067	0.797	0.799	0.969
PMOS	$1,25(OH)_2D_3$	MMP12	-0.262	0.309	0.308	0.837
PMOS	$1,25(OH)_2D_3$	MMP1	-0.071	0.787	0.789	0.969
PMOS	$1,25(OH)_2D_3$	MMP13	0.244	0.346	0.373	0.881
PMOS	$1,25(OH)_2D_3$	MMP14	-0.464	**0.061**	0.054	0.502
PMOS	$1,25(OH)_2D_3$	MMP16	-0.203	0.435	0.423	0.904
PMOS	$1,25(OH)_2D_3$	MMP10	-0.197	0.448	0.451	0.904
PMOS	$1,25(OH)_2D_3$	MMP8	0.060	0.820	0.819	0.969
PMOS	$1,25(OH)_2D_3$	TIMP1	0.012	0.963	0.963	0.969
PMOS	$1,25(OH)_2D_3$	TIMP2	-0.034	0.898	0.898	0.969
PMOS	$1,25(OH)_2D_3$	TIMP3	-0.221	0.394	0.398	0.884
PMOS	$1,25(OH)_2D_3$	MMP14/TIMP2	-0.470	**0.057**	0.057	0.502
PMOS	$1,25(OH)_2D_3$	MMP16/TIMP2	-0.156	0.549	0.549	0.922
PMOS	$1,25(OH)_2D_3$	MMP17/TIMP2	0.087	0.739	0.742	0.969
PMOS	$1,25(OH)_2D_3$	MMP14/TIMP3	-0.081	0.758	0.761	0.969
PMOS	$1,25(OH)_2D_3$	MMP16/TIMP3	0.252	0.329	0.323	0.863
PMOS	$1,25(OH)_2D_3$	MMP17/TIMP3	0.374	0.140	0.136	0.690
PMOS	$25(OH)D_3$	MMP9	0.014	0.955	0.955	0.969
PMOS	$25(OH)D_3$	MMP3	0.012	0.959	0.960	0.969
PMOS	$25(OH)D_3$	MMP7	0.030	0.899	0.900	0.969
PMOS	$25(OH)D_3$	MMP17	0.440	**0.052**	0.052	0.502
PMOS	$25(OH)D_3$	MMP2	0.215	0.362	0.365	0.884
PMOS	$25(OH)D_3$	MMP12	0.130	0.585	0.585	0.927
PMOS	$25(OH)D_3$	MMP1	-0.153	0.519	0.525	0.922
PMOS	$25(OH)D_3$	MMP13	0.109	0.646	0.664	0.969
PMOS	$25(OH)D_3$	MMP14	-0.125	0.599	0.628	0.932
PMOS	$25(OH)D_3$	MMP16	0.397	**0.083**	0.084	0.539
PMOS	$25(OH)D_3$	MMP10	-0.071	0.766	0.767	0.969
PMOS	$25(OH)D_3$	MMP8	0.267	0.256	0.259	0.827
PMOS	$25(OH)D_3$	TIMP1	0.009	0.969	0.970	0.969
PMOS	$25(OH)D_3$	TIMP2	0.054	0.820	0.814	0.969
PMOS	$25(OH)D_3$	TIMP3	-0.601	**0.005**	0.005	0.142
PMOS	$25(OH)D_3$	MMP14/TIMP2	-0.180	0.447	0.447	0.904

PMOS	$25(OH)D_3$	MMP16/TIMP2	0.174	0.463	0.479	0.904
PMOS	$25(OH)D_3$	MMP17/TIMP2	0.345	0.136	0.134	0.690
PMOS	$25(OH)D_3$	MMP14/TIMP3	0.311	0.181	0.186	0.726
PMOS	$25(OH)D_3$	MMP16/TIMP3	0.539	0.014	0.017	0.219
PMOS	$25(OH)D_3$	MMP17/TIMP3	0.600	**0.005**	0.006	0.142
PMOS	$24,25(OH)_2D_3$	MMP9	0.026	0.914	0.912	0.969
PMOS	$24,25(OH)_2D_3$	MMP3	-0.020	0.935	0.946	0.969
PMOS	$24,25(OH)_2D_3$	MMP7	0.036	0.881	0.873	0.969
PMOS	$24,25(OH)_2D_3$	MMP17	0.419	**0.066**	0.068	0.502
PMOS	$24,25(OH)_2D_3$	MMP2	0.204	0.387	0.385	0.884
PMOS	$24,25(OH)_2D_3$	MMP12	0.149	0.531	0.536	0.922
PMOS	$24,25(OH)_2D_3$	MMP1	-0.092	0.699	0.707	0.969
PMOS	$24,25(OH)_2D_3$	MMP13	0.038	0.874	0.870	0.969
PMOS	$24,25(OH)_2D_3$	MMP14	0.110	0.646	0.664	0.969
PMOS	$24,25(OH)_2D_3$	MMP16	0.381	0.098	0.102	0.548
PMOS	$24,25(OH)_2D_3$	MMP10	-0.033	0.892	0.892	0.969
PMOS	$24,25(OH)_2D_3$	MMP8	0.301	0.197	0.197	0.751
PMOS	$24,25(OH)_2D_3$	TIMP1	0.094	0.694	0.688	0.969
PMOS	$24,25(OH)_2D_3$	TIMP2	0.041	0.863	0.859	0.969
PMOS	$24,25(OH)_2D_3$	TIMP3	-0.585	**0.007**	0.007	0.142
PMOS	$24,25(OH)_2D_3$	MMP14/TIMP2	0.051	0.831	0.836	0.969
PMOS	$24,25(OH)_2D_3$	MMP16/TIMP2	0.156	0.510	0.501	0.922
PMOS	$24,25(OH)_2D_3$	MMP17/TIMP2	0.317	0.173	0.177	0.726
PMOS	$24,25(OH)_2D_3$	MMP14/TIMP3	0.451	0.046	0.049	0.502
PMOS	$24,25(OH)_2D_3$	MMP16/TIMP3	0.532	0.016	0.015	0.219
PMOS	$24,25(OH)_2D_3$	MMP17/TIMP3	0.590	**0.006**	0.006	0.142
